# Is the serum level of salusin-β associated with hypertension and atherosclerosis in the pediatric population?

**DOI:** 10.1007/s00467-014-2960-y

**Published:** 2014-09-23

**Authors:** Urszula Kołakowska, Elżbieta Kuroczycka–Saniutycz, Anna Wasilewska, Witold Olański

**Affiliations:** 1 Department of Pediatric Emergency Medicine, Medical University of Białystok, Białystok, Poland; 2 Department of Pediatrics and Nephrology, Medical University of Białystok, Waszyngtona 17, 15-274 Białystok, Poland

**Keywords:** Salusin-β, Essential hypertension, Atherogenic index, Children, Adolescents

## Abstract

**Background:**

Salusins are recently identified endogenous bioactive peptides that have hypotensive and bradycardiac effects. Salusin-β is involved in the pathogenesis of human atherosclerosis.

**Methods:**

This was a prospective cohort study of a young patient population with hypertension (HTN). Based on ambulatory blood pressure monitoring (ABPM), the adolescents were categorized into two groups, namely, a hypertensive group consisting of patients with essential (primary) HTN (HTN group) and a group consisting of patients with white-coat HTN [reference (R) group]. Correlations between serum salusin-β level and clinical, laboratory and ambulatory blood pressure (BP) variables were assessed.

**Results:**

The median salusin-β concentration was significantly higher in patients with essential HTN than in those with white-coat HTN (R group). Salusin-β was positively correlated with the body mass index *Z*-score, systolic and diastolic blood pressure (BP) from three independent measurements, mean systolic BP during the daytime, triglyceride (TG) level, and atherogenic index (TG/high-density lipoprotein-cholesterol ratio).

**Conclusions:**

The results of this preliminary study suggest that salusin-β may play an important role in the pathogenesis of HTN in a young population. Further research should focus on the role of salusin-β in the mechanism of essential HTN and the assessment of possible correlations between salusin-β and other well-known markers of atherosclerosis in both teenagers and adults. This research should serve as a base for future studies in this field.

## Introduction

Salusins are recently identified endogenous bioactive peptides which were first discovered by bioinformatic analysis of a full-length complementary DNA library [1]. Human salusin-α and -β are related peptides of 28 and 20 amino acids, respectively, produced from the same precursor, prosalusin [2]. It has been confirmed that salusins exist in human plasma in their originally predicted forms [3, 4], indicating their possible role as peptide hormones in humans [2]. They represent a new class of bioactive peptides with hypotensive and bradycardiac effects, among which salusin-β is the most potent. Salusins are multifunctional regulators of hemodynamics [1]. The role of salusins in hypertension (HTN) and atherosclerosis has not yet been clearly established.

Salusin-α may be involved in the pathogenesis of essential (primary) HTN [5], and salusin-β, which is theoretically synthesized concomitantly with salusin-α, causes profound hypotension without exerting an appreciable vasodilatory effect [5]. The hypotensive effect of salusins is achieved partly through activation of the parasympathetic nervous system [1], and elevated sympathetic nervous system activation has been shown in HTN [5]. Chronic increased sympathetic responsiveness may lead to essential HTN [5]. Salusin-β stimulates the release of arginine-vasopressin (AVP) and oxytocin from the rat neurohypophysis [6, 7]. Bolus injection of salusin-β induces rapid and temporary hypotension and bradycardia via a cholinergic mechanism and has direct negative inotropic effects; however salusin-β has no vasodilatory effect [1]. To the contrary, salusin-β induces potent hypotension via negative inotropic and chronotropic actions [2]. Circulating levels of salusin-β are standardly higher than those of salusin-α, but patients with essential HTN show significant but slightly lower levels than healthy volunteers [8]. Salusin-α, on the other hand, shows distinctly lower levels in essential HTN [8], which is a common risk factor for cardiovascular disease [5].

Atherosclerosis, which is often observed in the early stage of essential HTN [5], is a pathological injury-to-response process that is initiated by early inflammatory responses of the vascular endothelial cells [9]. HTN-induced mechanical stimuli, such as pressure overload, stretch, and shear stress, cause arterial endothelial injury, leading to the development of atherosclerosis [2]. Vasoactive agents, such as angiotensin II, urotensin II, and serotonin, all of which are agonists of G-protein–coupled receptors, play key roles as mediators linking HTN and atherosclerosis [10]. Salusins play an important role as endogenous modulators of atherogenesis [8]. Salusin-β exerts systemic proatherogenic activity, while salusin-α has a contrasting anti-atherogenic effect [8]. These two peptides induce opposite effects on macrophage foam cell formation [11]. Salusin-α suppresses foam cell formation, while salusin-β stimulates it [11]. In contrast to salusin-β, salusin-α has marginal mitogenic effects in vascular smooth muscle cells [1] and fibroblasts [2]. Reduced levels of serum salusin-α could be a reliable biomarker for detecting cardiovascular disease rather than increased salusin-β levels [8].

Salusins may be involved in both the maintenance of hemodynamic homeostasis and the regulation and development of human atherosclerosis [2].

The aim of this study was to test the hypothesis that serum salusin-α and salusin-β levels differ in adolescents with essential HTN compared to those of a reference group. An additional aim was to investigate the correlations between salusin-α, salusin-β and clinical, laboratory, and blood pressure (BP) monitoring variables.

## Patients and methods

This was a prospective cohort study of children and adolescents with essential (primary) HTN. The study cohort comprised 88 children (33 girls, 55 boys) aged 6–18 years who had been referred to the Department of Pediatrics and Nephrology, The Medical University of Białystok, Poland, between July 2010 and March 2014 by general practitioners. The majority of patients had elevated BP and were hospitalized to confirm or rule out the diagnosis of HTN.

Based on ambulatory blood pressure monitoring (ABPM) we categorized the children and adolescents into two groups. One group comprised 58 patients with essential HTN (43 boys, 15 girls; age range 6–18 years), and the second group was a reference (R) group consisting of 30 patients (12 boys, 18 girls; age range 7–17.5 years) with white-coat HTN.

Inclusion criteria were: age between 6 and 18 years; essential arterial HTN verified by ABPM as mean daytime and nighttime systolic BP (SBP) levels of ≥95th percentile for age, sex, and height, and a load SBP (LSBP) or load diastolic BP (LDBP) of >25 % [12]; normal levels of creatinine. Exclusion criteria were secondary forms of HTN; renal or hepatic dysfunction; heart failure; diabetes mellitus; hematological disease; systemic inflammatory conditions; autoimmune diseases; girls on contraceptive pills; subjects treated with hypertensive agents and medications known to affect BP values.

In the patients placed in the R group, essential HTN was excluded during the medical examination on the basis of ABPM (mean daytime and nighttime SBP and DBP levels of <95th percentile for age, sex, and height and a LSBP and LDBP of <25 %). Patients in the R group were not receiving any medication at the time of the examination, and their family history was negative for essential HTN or other cardiovascular diseases, diabetes, and metabolic syndrome.

For all subjects, careful clinical histories were taken, and physical examinations were performed. Height was measured in duplicate (when the between measurements exceeded 4 mm, a third measurement was taken) using a pediatric wall-mounted stadiometer, with the patient in a standing position with no shoes. Body weight was recorded with the patient wearing only lightweight underwear to the nearest 0.05 kg, using a digital medical scale. The body mass index (BMI) was calculated as weight (in kilograms) divided by the square of height (meters squared). The exact age of each patient was calculated from birth and examination dates. Age- and height-specific reference values for BMI and height were generated by the least mean squares method [13], which characterizes the distribution of a variable by its median (M), the coefficient of variation (S, i.e., the ratio of the standard deviation and mean), and skewness (L) required to transform the data to normality. Evaluation of these parameters was obtained by a maximum-likelihood curve-fitting algorithm to the original data plotted over the independent variable. The formula for calculating the *Z*-score of BMI or height was: LMS – SDS = {[Y/M (t)] L (t)-1}/[L (t) x S (t)], where SDS in the standard deviation score, Y is the individual observation, and L (t), M (t), and S (t) are the specific values of L, M, and S, respectively, interpolated for the child’s age and gender. The LMS values were taken from the OLAF study published by Kulaga et al. [14].

After 12 h of overnight fasting, blood samples were taken from each patient for the measurement of salusin-α and -β levels, morphology of the peripheral blood, basal glucose level, lipid profile, and serum creatinine, urea, and uric acid levels. The serum salusin-α and -β levels were measured using a commercially available ELISA kit (USCN Life Science Inc., Houston, TX) according to the manufacturer’s instructions. In brief, this assay employs the competitive inhibition enzyme immunoassay technique. A competitive inhibition reaction was launched between biotin-labeled salusin-α or -β and unlabeled salusin-α or -β, with the pre-coated antibody specific to salusin-α or -β, respectively. Next, avidin conjugated to horseradish peroxidase was added to each microplate well, and the intensity of color of each sample after the “stop” reaction was measured at 450 nm by a microtiter plate reader and compared with a standard curve. Serum salusin-β levels were expressed in picograms per milliliter, but the serum salusin-α level was lower than the method detection level (sensitivity) and therefore undetectable. Serum creatinine was determined by the Jaffé reaction, and uric acid was measured using a Hitachi apparatus (Hitachi, Chiyoda, Japan). The morphology of the peripheral blood was assessed on a Coulter analyzer MAXM (Beckman Coulter, Brea, CA). Serum cholesterol, high-density lipoprotein (HDL)-cholesterol, and triglycerides (TG) were determined by the enzymatic method using a Hitachi model 912 apparatus. Serum glucose was measured with the Integra 800 analyzer (Roche Diagnostics, Mannheim, Germany). ABPM was performed using the SpaceLabs Medical oscillometric blood pressure monitor (SpaceLab, Redwood, WA). The monitors were programmed to measure BP every 15 min during the daytime (8 a.m. to 10 p.m.) and every 30 min during the nighttime (10 p.m. to 8 a.m.); however, the periods were corrected according to the subjects’ diaries. Recording started between 8 and 9 a.m. and lasted for 24 h. Recordings with a minimum 80 % of measurements and without breaks of >2 h were considered to be sufficient for analysis. The mean SBP and DBP were calculated separately for the 24-h period and for the awake and asleep periods. We also analyzed the LSBP and LDBP during the day and night. HTN diagnosed on the basis of ABPM was defined as the mean daytime or nighttime SBP or DBP of ≥95th percentile and LSBP or LDBP daytime or nighttime levels of >25 % [12]. The values were adjusted by gender and body height according to the reference values provided by Wühl et al. [15]. Each subject or his guardian was asked to record the bedtime and time of awakening. After 24 h, the cuff and monitor were removed and the data downloaded using the manufacturer’s software.

The protocol was approved by the Bioethics Committee of The Medical University of Białystok in accordance with the Declaration of Helsinki. Informed consent was obtained from parents or guardian of all participants and children older than 16 years.

## Statistical methods

Data analysis was performed using the computer program Statistica 10.0 PL (StatSoft, Tulsa, OK). Discrete variables were expressed as counts (percentage), whereas continuous variables were expressed as the median and range, unless stated otherwise. The two groups were compared using the Mann–Whitney test for data not distributed normally. Correlations between salusin-β and other variables (clinical and laboratory parameters) were evaluated using standard methods, such as the Spearman’s test. The value of *p* < 0.05 was considered to be statistically significant.

## Results

Our study was conducted among 88 children and adolescents who were divided into two groups based on the diagnosis of essential (primary) HTN or not (white-coat HTN). The demographic, clinical, and and ambulatory BP data for each group are summarized in Table 1.

**Table 1:** Anthropometric, clinical, and metabolic characteristics of the study cohort according to the diagnosis, or not, of essential (primary) hypertension^a^

Anthropometric, clinical, and metabolic characteristics	HTN group (*n* = 58)	R group (*n* = 30)	*p*
Sex (M/F)	43/15	12/18	–
Age (years)	16 (13.6–17)	15 (12.6–17)	NS
BMI *Z*-score	1.55 (0.88–2.11)	0.38 (−0.25 to 0.84)	<0.01
Mean SBP (mmHg)^b^	134 (127.2–139)	115.5 (110–123)	<0.01
Mean DBP (mmHg)^b^	76 (72.5–85)	64.5 (61–70)	<0.01
Glucose (mg/dL)	90 (85–95)	90.5 (83.2–93.7)	NS
Cholesterol (mg/dL)	172 (146.2–194)	162 (142–189)	NS
Triglyceride (mg/dL)	100 (66–137)	80 (67–88)	<0.05
HDL-cholesterol (mg/dL)	52 (43–62)	46 (40.5–62)	NS
Triglyceride/HDL-cholesterol ratio	1.78 (1.11–2.83)	1.85 (0.88–2.10)	NS
Creatinine (mg/dL)	0.72 (0.57–0.86)	0.61 (0.48–0.73)	<0.01
Uric acid (mg/dL)	5.91 (5.03–6.71)	4.55 (3.57–5.57)	<0.01
Salusin-β (pg/mL)	446.43 (280.87–631.3)	350.3 (256.4–434.3)	<0.05

Values are presented as the median with the interquartile range (IQR: Q1–Q3) in parenthesis

M, Male; F, female; BMI *Z*-score, body mass index age- and height-specific reference; SBP, systolic blood pressure; DBP, diastolic blood pressure; HDL, high-density lipoprotein; NS, Not significant

^a^The group of patients diagnosed with essential (primary) hypertension (HTN) were classified into the HTN group; the subjects diagnosed with white-coat HTN were classified into the reference ®) group

^b^Mean of three readings/measurements

The median age did not differ between groups. The majority of patients in both HTN groups were male, which is in line with available reports in this area [16]. More boys than girls were diagnosed with essential HTN and placed in the HTN group [43/58 (74.1 %) vs.15/58 (25.9 %), respectively], whereas girls were in the majority in the R group (18/30, 60 %). The median BMI *Z*-score in the HTN group was 1.55 [interquartile range (IQR) 0.88–2.11] and statistically significantly higher than the median BMI *Z*-score of the reference group (*p* < 0.01). Of the 58 patients in the HTN group, 37 teenagers (63.8 %) were classified as overweight or obese. It should be noted that there were clear differences between the two study groups in that the hypertensive group (HTN) consisted predominantly of adolescent boys with a high BMI *Z*-score while the R group included many slender girls with white-coat HT.

A comparison of both groups revealed that the patients in the HTN group had higher serum levels of TG, Cr, and uric acid. We found no significant differences in plasma glucose, total cholesterol- and HDL-cholesterol concentrations between both groups. Data from the 24-h ABPM are shown in Table 2.

**Table 2:** Ambulatory blood pressure monitoring data

Parameters	HTN ( (*n* = 58)	R ( (*n* = 30)	*p*
Mean SBP/24 h (mmHg)	132 (127–137)	112 (110–114)	<0.05
Mean DBP/24 h (mmHg)	72 (67–75)	64.5 (63.7–65.2)	NS
Mean SBP/D (mmHg)	134 (129–140)	114 (111–116)	<0.05
Mean SBP/N (mmHg)	120 (112–125)	104 (102–105)	<0.05
Mean DBP/D (mmHg)	62 (58–66)	67 (67–67)	NS
Mean DBP/N (mmHg)	62.72 (49–90)	58.5 (56–60)	NS
LSBP (%) D	58.5 (44–73.2)	8.65 (5.8–11.4)	<0.05
LSBP (%) N	50 (23–80)	8.35 (4.1–12.5)	NS
LDBP (%) D	15.1 (6.8–24)	3.05 (1.5–4.5)	NS
LDBP (%) N	11 (0–27)	13.5 (6.7–20.2)	NS
SBP N drop (%)	11.8 (9–15.1)	8.85 (8.2–9.4)	NS
DBP N drop (%)	14.7 (9.9–20.5)	12.6 (9.2–16)	NS

Values are presented as the median with the IQR in parenthesis

LSBP/LDBP, load of SBP/DBP, respectively; D, day; N, night

Following the data presented in Table 2, we noticed significantly higher values of mean SBP and DBP (3 independent measurements), mean SBP during 24 h of ABPM and separately during the daytime and nighttime, and mean LSBP (%) during daytime.

**Fig. 1:**
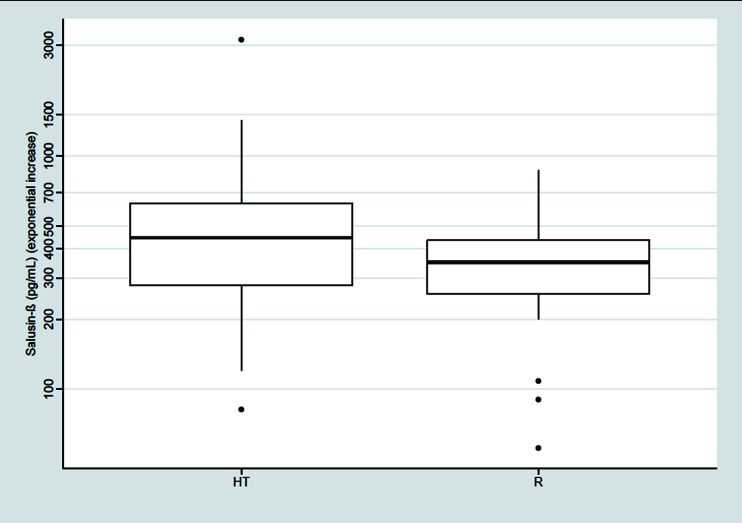
Comparison of salusin-β serum level (exponential increase) between the hypertensive subjects (*HT*) and the reference (*R*) subjects with white-coat HTN (*p* < 0.05). *Thick black horizontal line* Median, *top and bottom of box* interquartile range (IQR: Q1–Q3), *filled circles* outliers, *whiskers* standard deviation (SD)

The median salusin-β concentration was significantly higher in patients with essential HTN than in the subjects of the R group [446.43 (IQR 280.87–631.3) vs. 350.3 (IQR 256.4–434.3) pg/mL, respectively; *p* < 0.05], as shown on Fig. 1. The serum level of salusin-ɑ in both groups was below the method detection level (sensitivity).

**Fig. 2:**
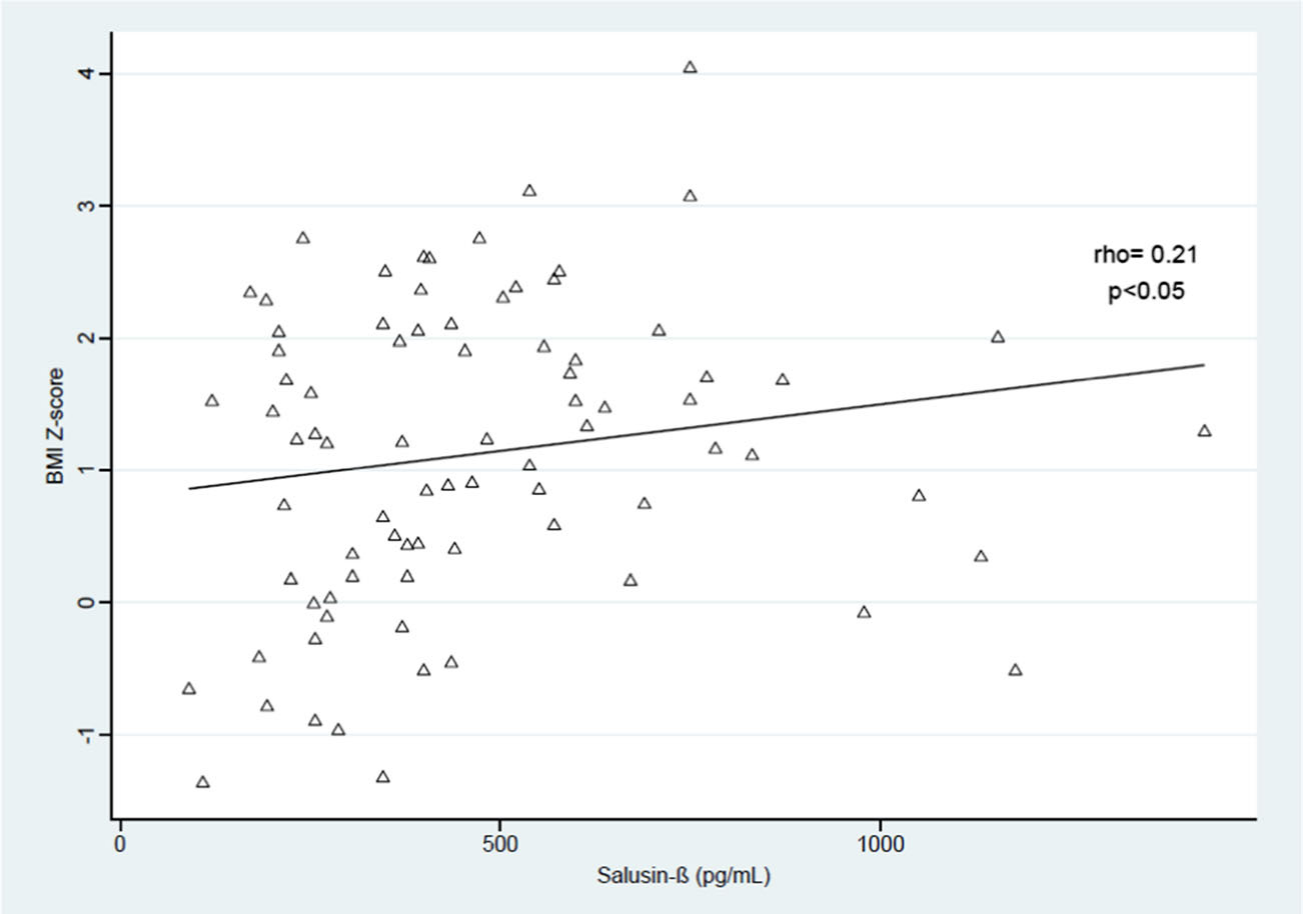
Correlation between serum salusin-β level and body mass index (*BMI*) *Z*-score. *BMI Z*-*score *BMI age- and height-specific reference

**Fig. 3:**
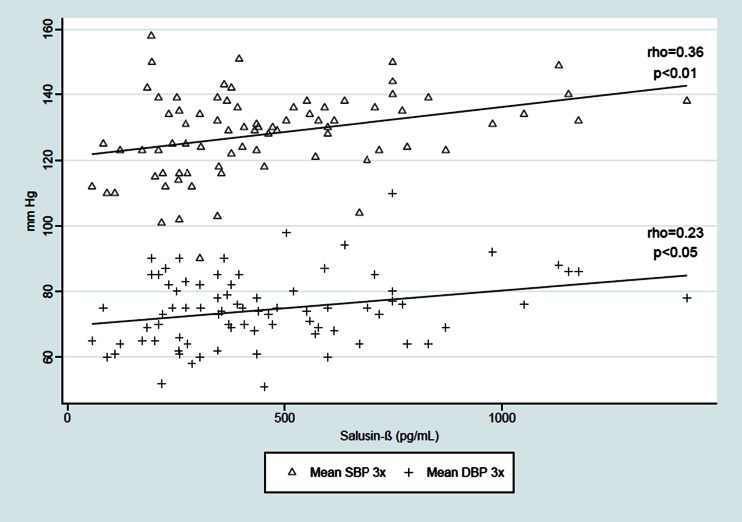
Correlation between serum salusin-β level and systolic and diastolic blood pressure (*BP*) (*SBP* and *DBP*, respectively) from three independent measurements. *3×* Three independent, duplicate measurements

**Fig. 4:**
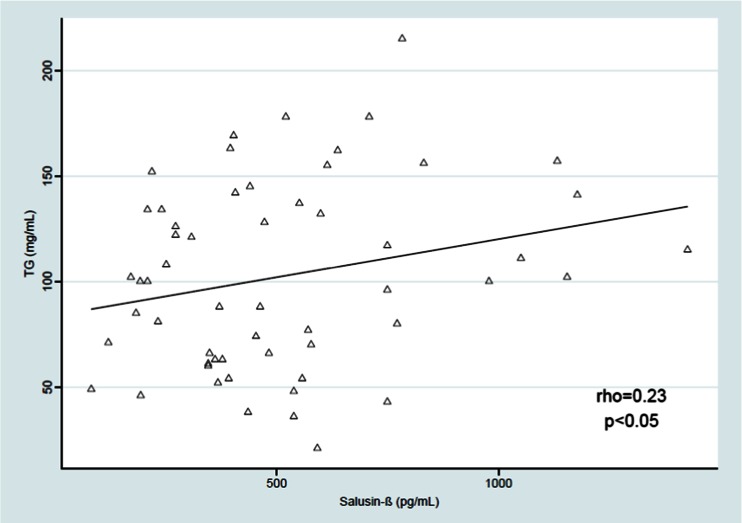
Correlation between serum salusin-β level and triglyceride (*TG*) content (rho = 0.23, *p* < 0.05)

**Fig. 5:**
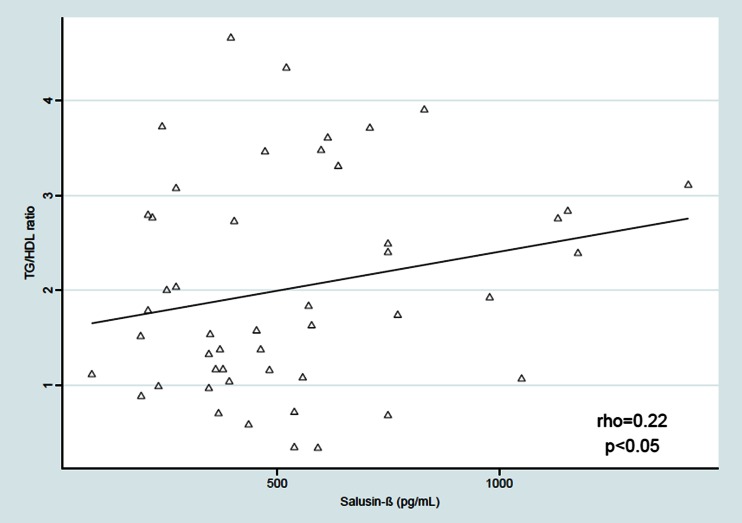
Correlation serum salusin-β level and TG/HDL ratio (rho = 0.22, *p* < 0.05). *HDL* High-density lipoprotein-cholesterol

Salusin-β was positively correlated with the BMI *Z*-score (rho = 0.23, *p* < 0.05) and with the SBP and DBP from three independent measurements [(rho = 0.36, *p* < 0.01;, rho = 0.23, *p* < 0.05), respectively]. The relationship between serum salusin-β level and mean SBP during the daytime was derived (rho = 0.26, *p* < 0.05), but we found no significant correlation between serum salusin-β level and daytime DBP, SBP and DBP during the nighttime and 24-h ABPM monitoring, LSBP and LDBP during the daytime and nighttime, and the SBP and DBP daytime and nighttime drop. We also found a positive relationship between salusin-β and TG level (rho = 0.23, *p* < 0.05) and between salusin-β and atherogenic index (TG/HDL-cholesterol ratio) (rho = 0.22, *p* < 0.05). The results are shown on Figs. 2–5.

## Discussion

In this study group, children and adolescents with essential (primary) HTN had a significantly higher serum salusin-β level than those assigned to the reference (R) group consisting of patients with white-coat HTN. We also found a positive correlation between the salusin-β blood level and the BMI *Z*-score, SBP, and DBP, based on three independent measurements, and between the mean SBP during 24-h ABPM and the daytime and nighttime separately, TG, and the TG/HDL ratio (atherogenic index). To our best knowledge this is the first clinical study which focuses on the association between serum salusin-β level and BP in children and adolescents. The serum salusin-ɑ level in our participants was below the method detection limit (sensitivity), and currently no published data on serum salusin-α levels are available in the pediatric patient population.

Salusins are considered to be multifunctional regulators of hemodynamics [1]. Serum salusin-α levels are significantly decreased in adult hypertensive patients relative to normotensive controls [5], and this peptide is associated with preclinical atherosclerotic markers in essential HTN [5]. Salusin-β acutely regulates hemodynamics and chronically induces atherosclerosis [17]. This peptide is expressed in the brain and various peripheral tissues [18] and is synthesized ubiquitously in human, rat, and mouse tissues, including the vasculature, central nervous system, and kidneys [1, 19, 20]. Salusin-β has also been shown to be present in human plasma and urine [4, 21, 22], indicating their possible role as peptide hormones in humans [7, 8, 23, 24]. Locally, salusins are synthesized in the fibroblast cells of the media in the aorta, but also in the smooth muscle cells of the media in the left internal mammary artery and saphena [25].

Very recent data indicate that neuroendocrine sources contribute only in a very limited manner to peripheral total salusin-β levels in humans [17]. However, because the major origin of total plasma salusin-β levels is not the neuroendocrine system, it should be under the control of as-yet unidentified systemic tissues/cells which produce and secrete the salusin-β peptide [17]. It was recently discovered that peripheral salusin-β levels are derived from non-pituitary sources [17]. Circulating salusin-β may be mostly bound to plasma proteins and thus devoid of potent hemodynamic activity. Circulating free salusin-β may function as a cardiotropic peptide hormone that modulates hemodynamics in humans [2, 6].

Sato et al. showed that the circulating level of salusin-β in human is significantly but slightly lower in patients with essential HTN than in healthy volunteers [8]. In this same study, circulating salusin-β levels increased in patients with coronary artery disease [8]. In our pediatric population the serum level of salusin-β was higher in the HTN group than in the R group, possibly due to the increased BMI and higher frequency of obesity in the patients of the HTN group.

BP is regulated by many factors, such as vascular structure, fluid volume, sympathetic nervous system, and the renin–angiotensin system (RAS) [26]. Salusin-β, when injected as a bolus, induces dose-dependent, rapid, and temporary hypotension and bradycardia via a cholinergic mechanism and also has direct negative inotropic cardiac effects and chronotropic actions without a vasodilatory effect [1, 2, 27]. Salusin-β–induced hypotension and bradycardia can be completely blocked by pretreatment with atropine, a muscarinic receptor antagonist, but not by propranolol [2, 27].

Our understanding of the mechanisms related to central neural regulation of BP has progressed rapidly in recent years [28]. Neuroendocrine salusin-β is localized in the vasopressin-expressing neurons of the rat posterior pituitary and hypothalamus, which has tempted many authors to speculate its neural secretion into the systemic circulation via axon terminals [1, 4, 7, 19, 20, 29]. Many studies have shown that salusin-β may participate in the regulation of BP in coordinated manner with arginine–vasopressin (AVP) [29]. Salusin-β stimulates the secretion of AVP from the rat neurohypophysis in vitro in a concentration-dependent manner [1] and coexists with AVP in the hypothalamo-neurohypophyseal system of the rat under normal conditions [29]. These findings suggest that salusin-β has the potential to act as a neuropeptide that regulates body fluid homeostasis via its modulation of AVP release [7]. Microinjection of salusin-β into the paraventricular nucleus (PVN) increases BP via the release of norepinephrine and AVP in renovascular hypertensive rats [8, 30]. This action is apparently different from the depressor response determined by intravenous administration of salusins. These unexpected findings may be explained by the observation that salusin-β level and the number of salusin-β-like immunopositive neurons in the rostral ventrolateral medulla (RVLM), which is a central vasomotor center that plays a critical role in the regulation of sympathetic outflow and BP [31], have been found to be greatly increased in hypertensive rats compared to normotensive rats [18]. A number of researchers have shown that salusin-β in the RVLM does not stimulate AVP release from both the RVLM and neurohypophysis, but rather activates the presympathetic neurons in the RVLM, which in turn increases renal sympathetic activity (RSNA), median arterial pressure (MAP) and heart rate (HR) [18]. Salusin-β has been observed to increase RSNA, MAP, and HR in a dose-related manner in model of hypertensive rats, but it failed to induce any significant effect in normotensive rats [18]. However, salusin-β in the PVN not only increases the plasma AVP level, which partially contributes to the pressor response, but also increases AVP release from the RVLM via the projection from PVN to RVLM, which in turn contributes to renal sympathetic activation and its related pressor effect and increased HR [18]. Increased activity of neurons in the RVLM contributes to experimental and essential HTN [28]. Overactivity of the sympathetic nervous system is one of the more important causes of essential HTN [32] and contributes to the pathogenesis of HTN and progression of organ damage [33]. There is a possibility that the salusins act on the central nervous system to decrease sympathetic nerve activity and arterial pressure [26]. Zhang et al. discovered that neither circulating AVP nor AVP in the RVLM is involved in the sympatho-excitatory and pressor effects of salusin-β in the RVLM [18]. There is also a possibility that the RAS may be involved in the pressor response to salusin-β in the RVLM because the brain RAS is known to be activated in this model of HTN [34–36].

Our results show that children with essential HTN had higher plasma salusin-β levels than children with white-coat hypertension enrolled in a reference group. It therefore seems possible that future comparisons of children with essential HTN and healthy children may reveal that this difference is more significant than the that found in our study. Unfortunately, no published data are currently available in this area, although intravenous salusin-β administration to intact anesthetized rats has been found to cause hypotension and bradycardia [1]. More research on this topic needs to be undertaken because the mechanism of this result is still unknown.

Evidence recently emerging from animal and human studies shows a clear association between novel salusin peptides and atherosclerosis [2], as well as the important roles of these peptides as endogenous modulators of atherogenesis [8]. Atherosclerosis is a complex and multifactorial disease whose pathogenesis is associated with inflammatory responses [37, 38]. It has been reported that the endogenous salusin-β excessively produced in vascular lesions could contribute to the development of atherosclerosis [2, 11, 39]. Salusin-β is released from human monocytes/macrophage, suggesting a possible autocrine/paracrine role in the development and progression of atherosclerosis [8, 22]. The development of atherosclerosis is influenced by abnormalities in cellular cholesterol homeostasis in subendothelial macrophages [12]. The TG)/HDL-cholesterol ratio has been reported to be a useful marker of atherogenic lipid abnormalities, insulin resistance, and cardiovascular disease [40, 41]. Consequently, the TG/HDL ratio may help identify children and adolescents at high risk for structural vascular changes and metabolic derangement [41]. In our study we derived a positive correlation between serum salusin-β level and the TG/HDL ratio. Additionally, we found higher salusin-β plasma levels in obese children which was positively correlated with the BMI *Z*-score. Unfortunately, there are currently no research results available in this area.

To summarize, our results, while preliminary, suggest that salusin-β, but not salusin alpha, may play an important role in the pathogenesis of HTN in the pediatric populations. However, we recommend that further research be undertaken to assess the role of salusin-β in the mechanism of essential HTN and the possible correlations between salusin-β and other well-known markers of atherosclerosis in both teenagers and adults. Our study will hopefully serve as a base for future studies in this field.

Finally, a number of important limitations need to be considered. First, the reference group was not a group of healthy teenagers, but a group of patients in whom HTN was not confirmed in the 24-h ABPM; consequently, we considered these subjects to belong to the white-coat HT group. Secondly, the two study groups clearly differed, with the hypertensive group (HTN) predominantly consisting of adolescent males, most of whom had a high BMI *Z*-score, and the reference group (R) primarily consisting of slender girls with white-coat HT.
